# Ichthyosis Skin Changes in a Patient With Hereditary Hemochromatosis

**DOI:** 10.7759/cureus.52823

**Published:** 2024-01-23

**Authors:** Neha Arora, Kaycee Nguyen, Andrew Hudson, Lindsay Bicknell

**Affiliations:** 1 Dermatology, Texas A&M School of Medicine, Baylor University Medical Center, Dallas, USA; 2 Medicine, Texas A&M School of Medicine, Baylor University Medical Center, Dallas, USA; 3 Dermatology, Baylor Scott & White Health, Temple, USA

**Keywords:** imbalance in iron homeostasis, ichthyosis, cutaneous manifestation, hereditary hemochromatosis, ichthyosis vulgaris

## Abstract

Hereditary hemochromatosis (HH) is characterized by elevated iron absorption in the body, leading to iron accumulation with subsequent dysfunction and end-organ damage. While the progression of the disease can result in arthralgias, hepatomegaly, cardiomyopathies, and diabetes, over a third of HH patients present with cutaneous manifestations. We present the case of a 56-year-old male with HH who presented to dermatology with a rash and diffuse scaling. The patient exhibited brown plate-like scales clinically consistent with diffuse ichthyosis vulgaris. While ichthyosis has been seen in patients with idiopathic hemochromatosis, its association with HH is not well reported. Due to the high prevalence of cutaneous involvement in hereditary hemochromatosis, physicians should familiarize themselves with ichthyosis and the other dermatologic manifestations of this disease.

## Introduction

Hereditary hemochromatosis (HH) is an autosomal recessive genetic disorder characterized by an imbalance in iron homeostasis. It is the most common inherited single-gene disorder in the American Caucasian population, with at least one in 10 people carrying the mutation [1]. HH is characterized by increased iron absorption and is primarily caused by a mutation in the *HFE* gene [1]. This mutation impairs the function of hepcidin, leading to pathologic increases in iron storage, which can deposit in tissues throughout the body and lead to systemic symptoms [1]. Since females naturally lose a portion of the excess iron through menstruation, pregnancy, and lactation, the affected males usually develop the disease manifestations earlier than females [2]. The clinical presentation of HH can be vague, and patients often report fatigue and arthralgias as early symptoms. However, the complications associated with this condition include hepatomegaly leading to cirrhosis, cardiomyopathies and dysrhythmias, diabetes, and symmetric arthropathies [2]. With early detection and treatment, the progression of the disease to the liver, heart, and endocrine glands occurs less frequently now, although dermatologic manifestations are still commonly reported.

## Case presentation

**Figure 1:**
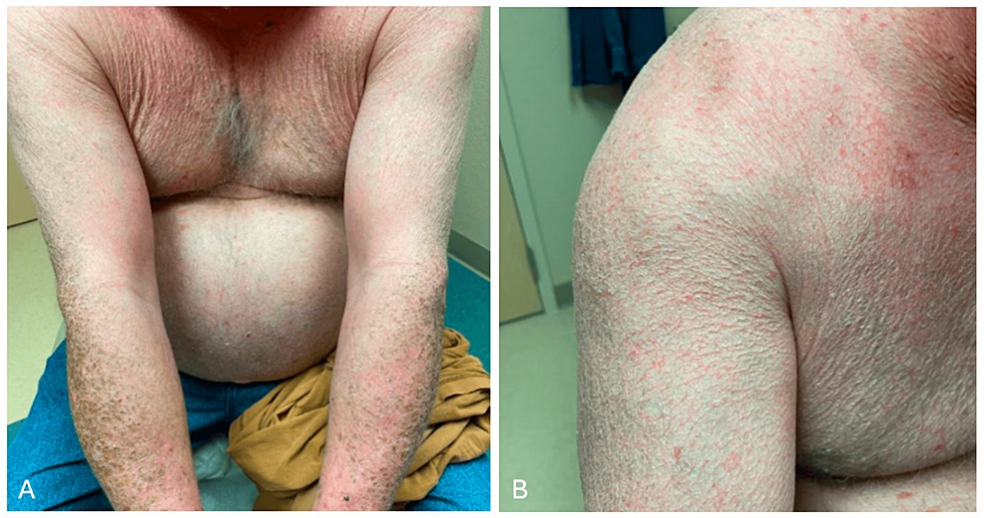
Brown scales present along the patient’s arms and forearms

**Figure 2:**
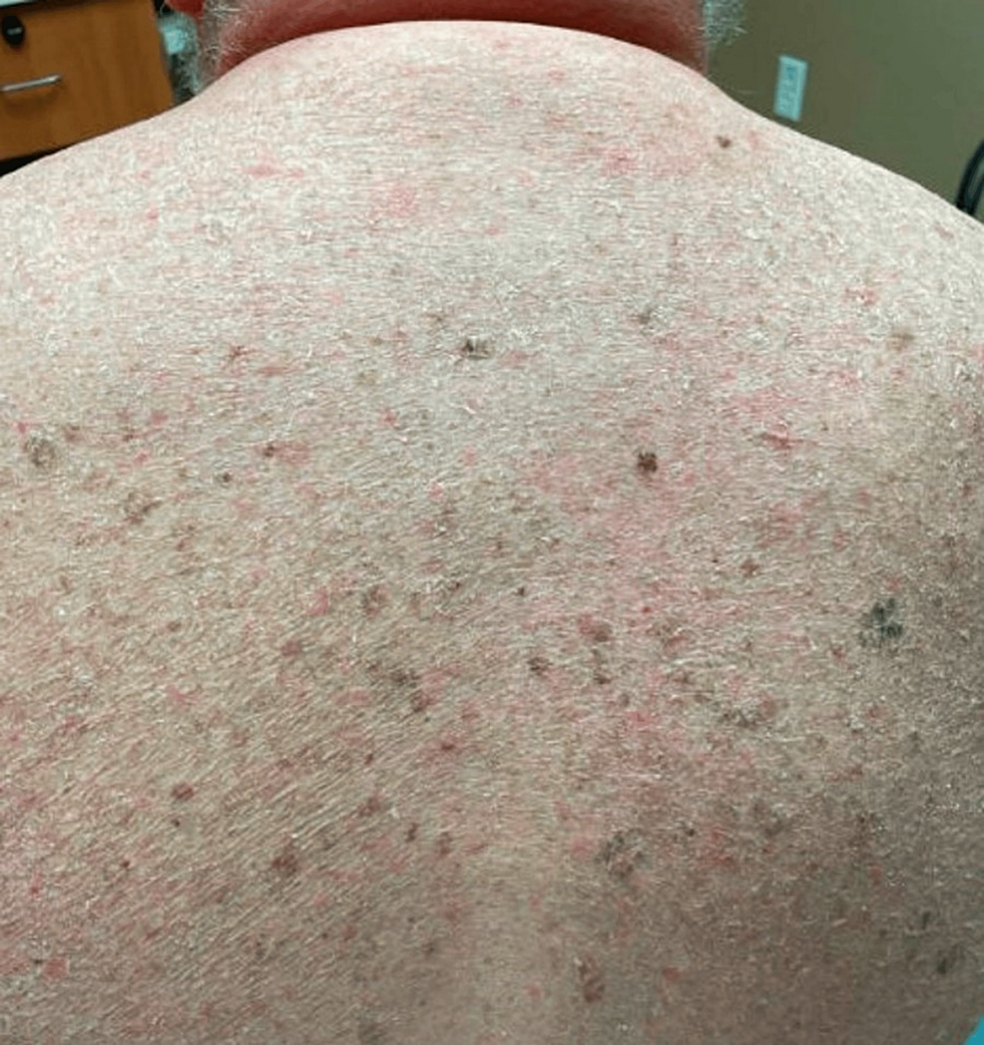
Diffuse scaling throughout the patient’s back

A 56-year-old male with hereditary hemochromatosis presented with a chronic rash with diffuse scaling throughout his arms (Figure 1), back (Figure 2), scalp, and ears. The patient’s arms exhibited plate-like, or “fish-like,” brown scales resembling diffuse ichthyosis vulgaris. The patient reported dryness and scaliness since childhood, and prior therapy included over-the-counter emollients with minimal relief.

The patient had been previously found to have compound heterozygosity for mutation in the *HFE* gene, confirming a diagnosis of HH. The patient’s laboratory results showed elevated hemoglobin, glycosylated hemoglobin (HbA1c), ferritin, total iron-binding capacity, and transferrin. The patient reported undergoing therapeutic phlebotomy for the management of HH, which did not impact the severity or distribution of the skin findings.

The patient was instructed to use urea cream and emollients containing lactic acid in all affected areas. Further genetic testing of family members was deferred at the time per patient preference and given its low likelihood of changing management. The patient reported improvement in dryness and scale six months following the initiation of treatment.

## Discussion

Approximately one-third of HH patients present with skin hyperpigmentation, which can be generalized but is more commonly observed on exposed skin [2]. The hyperpigmentation can appear gray due to dermal iron accumulation (described as “slate-gray skin”) or brown from increased melanin production [3,4]. Less frequently, the mucous membranes and conjunctiva may also be affected [3]. While skin hyperpigmentation is the most prevalent cutaneous finding of HH, various other dermatologic manifestations have been reported.

Ichthyosis describes a group of skin conditions characterized by impaired skin barrier integrity leading to transepidermal water loss and epidermal hyperproliferation [5]. This subsequently results in the appearance of fish-like scales and skin flaking. Ichthyosis disorders can be inherited or acquired in the setting of malignancy, infection, and inflammatory or autoimmune disorders [5].

Although ichthyosis in HH patients is not often discussed, cases of patients with idiopathic hemochromatosis presenting with ichthyosis-like skin changes have been documented [6]. Clinical presentation ranges from xerosis to generalized ichthyosis vulgaris, with the most commonly affected areas being the forearms, wrists, and feet [6]. Although not the case for our patient, typically, ichthyotic changes worsen with exacerbations of hemochromatosis but are alleviated with phlebotomy [6]. Additional dermatologic manifestations can include skin atrophy with hyperkeratosis predominantly in the pretibial region, palmar erythema, spider angiomas, lipodystrophy, and alopecia [6]. The loss of body hair and koilonychia may occur, but these changes are often not reversible even after treatment [6]. Our patient did not present with any of the additional cutaneous findings listed above.

The first-line treatment for ichthyosis includes creams and ointments containing agents such as salt, urea, or glycerol to increase the skin’s water-binding capacity [7]. Topical keratolytic formulations with alpha-hydroxy acids, salicylic acid, and high-dose urea can also be used in combination with retinoids to promote skin cell turnover and prevent hyperproliferation [7]. Phlebotomy can be considered in patients with ichthyosis refractory to standard treatments [7].

Interestingly, the patient’s intermittent phlebotomy for the management of HH had not improved his ichthyosis. The treatment plan included first-line agents including urea cream for improved hydration and emollients containing lactic acid for its keratolytic properties. After six months, the patient reported improvement in ichthyosis findings with treatment.

## Conclusions

Hereditary hemochromatosis (HH) can present with a wide variety of symptoms including musculoskeletal findings, endocrine dysregulation, liver disease, and skin hyperpigmentation. Here, we present the case of a 56-year-old male with hereditary hemochromatosis who exhibits diffuse ichthyosis vulgaris. Given the high prevalence and potential clinical significance of skin manifestations in HH, physicians should familiarize themselves with the varied cutaneous presentations of hereditary hemochromatosis, including ichthyosis vulgaris.
